# *Fli-1* Overexpression in Hematopoietic Progenitors Deregulates T Cell Development and Induces Pre-T Cell Lymphoblastic Leukaemia/Lymphoma

**DOI:** 10.1371/journal.pone.0062346

**Published:** 2013-05-07

**Authors:** Monique F. M. A. Smeets, Angela C. Chan, Samantha Dagger, Cara K. Bradley, Andrew Wei, David J. Izon

**Affiliations:** 1 Haematology and Leukaemia Unit, St. Vincent’s Institute, University of Melbourne, Fitzroy, Victoria, Australia; 2 Department of Microbiology and Immunology, University of Melbourne, Parkville, Victoria, Australia; 3 School of Pathology and Laboratory Medicine, University of Western Australia, Crawley, Western Australia, Australia; 4 Gennea, Sydney, New South Wales, Australia; 5 Department of Clinical Haematology, The Alfred Hospital and The Australian Centre for Blood Diseases, Monash University, Melbourne, Victoria, Australia; Purdue University, United States of America

## Abstract

The *Ets* transcription factor *Fli-1* is preferentially expressed in hematopoietic tissues and cells, including immature T cells, but the role of *Fli-1* in T cell development has not been closely examined. To address this we retrovirally overexpressed *Fli-1* in various *in vitro* and *in vivo* settings and analysed its effect on T cell development. We found that *Fli-1* overexpression perturbed the DN to DP transition and inhibited CD4 development whilst enhancing CD8 development both *in vitro* and *in vivo*. Surprisingly, *Fli-1* overexpression *in vivo* eventuated in development of pre-T cell lymphoblastic leukaemia/lymphoma (pre-T LBL). Known *Fli-1* target genes such as the pro-survival *Bcl-2* family members were not found to be upregulated. In contrast, we found increased NOTCH1 expression in all *Fli-1* T cells and detected *Notch1* mutations in all tumours. These data show a novel function for *Fli-1* in T cell development and leukaemogenesis and provide a new mouse model of pre-T LBL to identify treatment options that target the *Fli-1* and *Notch1* signalling pathways.

## Introduction

The Friend Leukemia Virus Integration 1 (*Fli-1*) is a member of the Ets family of transcription factors [1]. *Fli-1* is expressed in hematopoietic lineages and vascular endothelial cells and regulates the expression of multiple target genes involved in proliferation, differentiation and cell death. Originally, *Fli-1* was discovered as a common retroviral insertion site in Friend Murine Leukemia Virus-induced erythroleukaemia and subsequently as a common rearrangement in human Ewing’s sarcoma resulting in an EWS/Fli1 fusion product [2]. *Fli-1* is essential for embryonic development and has been shown to be required for megakaryocyte development as well as play a major role in myeloid, erythroid and natural killer (NK) cell development [1], [3], [4], [5]. It has also been demonstrated that *Fli-1* is expressed in immature T cells and concomitantly downregulated in pre-B cells [6]. Interestingly, *H-2K^K^-Fli-1* transgenic mice, which express high levels of FLI-1 in the thymus and spleen, die of an immunological renal disease and display increased numbers of mature B cells with reduced activation-induced cell death but no significant difference in CD4^+^CD8^+^ T cell distribution [7]. The exact role of *Fli-1* in T cell development is therefore not clear.

T cell development initiates when a blood-borne foetal liver (FL) or bone marrow (BM) precursor enters the thymus [8]. These are termed double negative (DN) cells, as they do not express CD4 or CD8. DN thymocytes undergo an ordered development based on the expression of CD44 and CD25. DN1 cells (CD25^−^44^+^) can reconstitute the T, B, dendritic cell (DC), and NK lineages [9], [10]. DN2 cells (CD25^+^44^+^) generate T cells, NK cells, DC and myeloid cells [10], [11], [12], [13]. TCRβ rearrangement occurs at the DN3 stage (CD25^+^44^−^) and this provides irrevocable commitment to the αβ T cell lineage [14]. Further development of αβ T cells requires signalling through the pre-TCR complex [15]. The pre-TCR is responsible for initiating the DN to DP transition [15]. Finally, DN4 cells (CD25^−^44^−^) are rapidly dividing blasts that spontaneously become CD4^+^8^+^ (double positive; DP) [16]. The DN to DP transition is marked by enormous proliferation [17]. Therefore, exquisite control is required at this particular checkpoint or oncogenesis may arise. DP cells are subjected to a series of stringent criteria that select for either mature CD4^+^8^−^ or CD4^−^8^+^ (single positive; SP) T cells that have moderate affinity for self-MHC but retain their ability to respond to foreign antigens [8].

We found that *Fli-1* overexpression perturbed the DN to DP transition *in vitro* as well as inhibited CD4 differentiation and promoted CD8 T cell development *in vitro* and *in vivo*. Interestingly, *Fli-1* overexpression *in vivo* eventually resulted in a fatal T cell lymphoblastic leukaemia/lymphoma with infiltration of leukaemic cells into the thymus, spleen, lymph node, bone marrow and liver. No enhancement of pro-survival *Bcl-2* family members was evident in *Fli-1* transduced cells, but subsequent analysis revealed NOTCH1 upregulation in all leukaemic *Fli-1* cells and the presence of 5′ *Notch1* deletions and PEST mutations.

## Design and Methods

This research was reviewed and approved by the St. Vincent’s Animal Ethics Committee. AEC# 012/10.

### Mice

All animal experiments were performed in accordance with the St. Vincent’s Hospital Animal Ethics committee. Either CD45.1 or CD45.2 C57BL/6 mice between 8–12 weeks old were used throughout this study. Mice were monitored daily and control and *Fli-*1 mice were sacrificed simultaneously by cervical dislocation when *Fli-1* mice became moribund.

### Antibodies

Antibodies used for surface staining included anti-TCR (H57) (PE), anti-CD4 (APC), anti-CD25 (PE), anti-CD44 (APC), and anti-CD8 (PerCP) (BD-Pharmingen, San Diego, CA, USA). Lineage depletion for CD25/44 staining was accomplished by staining cells with a cocktail of biotinylated mAb (anti-CD3, CD4, CD8, B220, Mac-1, Gr-1, Ter119 and NK1.1; (BD-Pharmingen, San Diego, CA, USA) and gating out SA-PeCy7 (BD-Pharmingen, San Diego, CA, USA).

### Flow Cytometry

Cell suspensions from mouse BM, thymus, spleen, lymph node or liver were washed in PBS with 2% FBS and 0.01% NaN_3_ (FACS buffer). Fc receptors were blocked with 2.4G2 (anti-FcR clone). Cells were then stained with primary antibodies for 20 min at 4°C and washed in 200 µl FACS buffer. Staining with streptavidin secondary reagents was performed in an identical manner. Labelled cells were run on a Becton Dickinson FACScalibur or LSR II, and analysed using FlowJo software (Treestar, Inc., San Carlos, CA). Routinely, 3×10^4^ to 2×10^5^ events were collected. Dead cells were excluded using FSC and SSC gates and with propidium iodide where possible.

Intracellular flow cytometry was performed by washing cells in 0.03% saponin (w/v) in FACS buffer (SAP FACS). Cells were then stained with anti-NOTCH1 (PE) or isotype control (PE) in SAP FACS and after washing, run on a FACScalibur or LSRII analysis. Routinely, 3×10^4^ to 2×10^5^ events were collected.

### Plasmids

MigR1-GFP was a gift from Warren Pear and was used as described previously [18]. *Fli-1* cDNA was cloned from 129Sv/J embryoid bodies, ligated into MigR1 and the insert confirmed by sequencing.

### Retroviral Transduction and Transplantation

Red cell depleted CD45.1 FL or BM cells from 5-fluorouracil-treated (100 mg/kg) mice were used as donor cells and transplanted into CD45.2 lethally irradiated (2×550 cGy) recipients. Up to 2 million E15.5 FL or adult BM cells were precultured for 24–48 hours in 2 ml complete IMDM with 50 µM β-mercaptoethanol, 50 ng/ml SCF, 6 ng/ml IL-3, 20 ng/ml IL-6, 4 ng/ml IL-1β and 1 ng/ml IFN-γ. Subsequently, 5×10^5^ FL/BM cells were centrifuged with 50% (v/v) retroviral supernatant at 1100 g for 90 min in 8 µg/ml polybrene or on Retronectin-coated plates (Takara Holdings, Inc. Shiga, Japan). One day later, lethally irradiated mice were injected intravenously with at least 2×10^5^ transduced FL/BM cells. Hematopoietic and lymphoid organs from transplanted mice were analysed at 6–12 weeks post-transplant or whenever they developed disease. Results from FL and BM transplanted mice were pooled for statistical analysis.

### Foetal Thymic Organ Culture

FL reconstitution of FTOCs was performed as described previously [18]. The reconstituted foetal thymic lobes were placed on 0.8 µm polycarbonate membranes (Isopore, Millipore, Ireland) floating on 2 ml complete IMDM and analysed by flow cytometry 14 days later.

### OP9-DL1 Co-cultures

E15 foetal liver (FL) cells were cultured on OP9-DL1 cells for 6 days in 5 ng/ml FLT3L and 0.25 ng/ml IL-7 [19]. FL cells were retrovirally transduced with either MigR1 control or *Fli-1*. Four days later the GFP^+^ cells were analysed for presence of DN1–4 progenitors by flow cytometry as described above.

### Histology

Fresh tissues (thymus, spleen, sternum) were fixed in Bouin’s fixative overnight and then placed into 70% ethanol and embedded in paraffin. Five µm sections were cut and, after standard histological procedure for dehydration, stained with haematoxylin and eosin.

### Western Blot Analysis

Cells were lysed in RIPA buffer (25 mM Tris-Cl [pH 7.6], 150 mM NaCl, 1% NP40, 1% sodium deoxycholate, and 0.1% SDS) with protease inhibitors (Complete Mini EDTA free protease inhibitor tablets, Roche Diagnostics, Castle Hill, NSW, Australia) and equal amounts (20 µg) of total protein per sample were separated via 10% SDS-PAGE and transferred to Immobilon-P (PVDF) transfer membrane (Millipore, North Ryde, NSW, Australia) for western blotting. Proteins were detected using primary antibodies against FLI-1 (sc-356, Santa Cruz, CA USA) and beta-ACTIN (A5316, Sigma-Aldrich, Castle Hill, NSW, Australia) and secondary HRP conjugated antibodies followed by visualisation using ECL reagents (Santa Cruz, CA USA).

### Southern Blot Analysis

The DIG High Prime DNA Labelling and Detection Starter Kit II (Roche Diagnostics, Castle Hill, NSW, Australia) was used for Southern blot assay. In brief, DNA (5 µg) was digested with *Eco*RI, size separated on a 0.8% agarose gel, and then capillary-transferred onto positively charged Nylon membranes (Roche Diagnostics, Castle Hill, NSW, Australia). A 1.4 Kb IRES-GFP fragment was purified, labelled with digoxigenin and used as a probe according to the manufacturer’s recommendations.

### TCRβ VDJ Rearrangement, *Notch1* Mutation and Deletion Analysis

TCRβ VDJ rearrangements as well as *Notch1* PEST mutations and 5′ deletions were examined by genomic PCR. Genomic DNA was isolated from total thymus or spleen and 100–125 ng was used per PCR reaction [20]. TCRβ VDJ rearrangements were analysed using the following primers: TCR Vbeta10 F 5′-GCGCTTCTCACCTCAGTCTTCAG and TCR Jbeta2 R 5′TGAGAGCTGTCTCCTACTATCGATT [21]. The PEST domain (exon 34) of murine *Notch1* was amplified with the primers PEST1F: 5′-TACCAGGGCCTGCCCAACAC and PEST2R: 5′-GCCTCTGGAATGTGGGTGAT [22]. Type 1 *Notch1* deletions were analysed using the primers: Notch1 P1 F: 5′- CCATGGTGGAATGCCTACTT and Notch1 P2 R: 5′- CGTTTGGGTAGAAGAGATGC and control primers G3PDH F: 5′- ACCACAGTCCATGCCATCAC and G3PDH R: 5′- TCCACCACCCTGTTGCTGTA [23], [24]. For sequence analysis DNA was amplified using a high fidelity polymerase. PCR products were then gel purified, sequenced and trace files were manually analysed**.**

### Quantitative Real-time PCR

Total RNA was extracted using the NucleoSpin RNA II kit (Macherey Nagel, Düren, Germany). First-strand cDNA synthesis was carried out with 1 µg of total RNA using random hexamers and MuMLV RT (New England Biolabs, Ipswich, MA USA). Real-time PCR was performed with a Rotor-Gene 3000 (Corbett Robotics, Brisbane, QLD, Australia) with SYBR Green for *Bcl-2*, *Bcl-xL* and *Mcl-1*. SYBR Green primer sets: *Bcl-2* F: 5′-CCGGGAGAACAGGGTATGATAA and *Bcl-2* R: 5′-CCCACTCGTAGCCCCTCTG, *Bcl-xL* F: 5′-TCTACGGGAACAATGCAGCA and *Bcl-xL* R: 5′-AGGAACCAGCGGTTGAAGC, *Mcl-1* F: 5′-TGGAGTTCTTCCACGTACAGGA and *Mcl-1* R: 5′-AGCAACACCCGCAAAAGC and *Gapdh* F: 5′-CATGTTTGTGATGGGTGTG and *Gapdh* R: 5′-CATGGACTGTGGTCATGAG. For *Fli-1* and *Notch1* the following Taqman Gene Expression Assays were used: Fli-1 Mm00484410_m1, Notch1 Mm00435245_m1 and Gapdh: Mm99999915_g1 (Applied Biosystems). Gene expression levels were calculated relative to *Gapdh* and data were normalized by dividing each expression value by the median of gene expression of MigR1 control mice.

### Statistics

Student t-tests were performed on data that was normally distributed whilst Mann-Whitney U tests were performed on data that was not normally distributed. These decisions were made using Instat 3.0 software (GraphPad Software, LaJolla, CA, USA). The Kaplan-Meier curve was generated using Prism (GraphPad Software, LaJolla, CA, USA) and significance for survival curve differences were calculated using the Log-rank test. Significance was determined as p<0.05 or less.

## Results

### *Fli-1* Overexpression Perturbs T cell Development *in vitro* and *in vivo*

**Figure 1:**
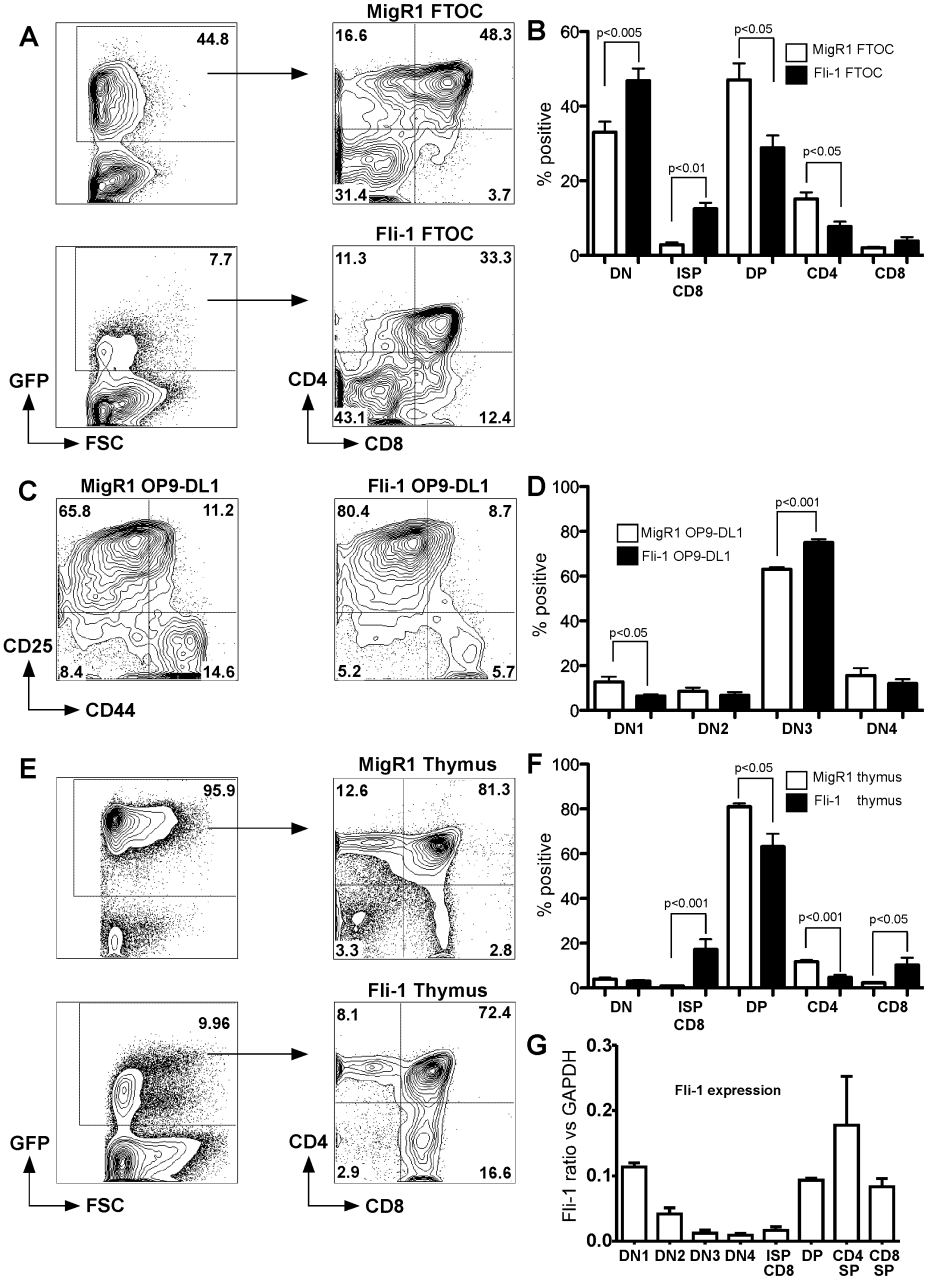
*Fli-1* overexpression perturbs T cell development *in vitro* and *in vivo*. A. FL cells were transduced with MigR1 or *Fli-1* and used to reconstitute foetal thymic lobes. Cultures were analysed 14 days later with anti-CD4 and CD8 by flow cytometry. Representative plot of at least 4 independent experiments. B. Graph of GFP^+^ FTOC MigR1 and *Fli-1* thymocyte populations. Data are represented as mean ± SEM; n = 4. C. MigR1 and *Fli-1* transduced FL cells grown on OP9-DL1 cells for 8 days were analysed for presence of DN1–4 by flow cytometry using Lin^−^, CD25 and CD44. Plot representative of 5 independent experiments. D. Graph of GFP^+^ DN cells of MigR1 and *Fli-1* transduced FL grown on OP9-DL1 cells. Data are represented as mean ± SEM; n = 5. E. BM cells were transduced with MigR1 or *Fli-1* and used to transplant lethally irradiated mice. Pre-leukaemic thymocytes were analysed 3 months later with anti-CD4 and CD8 by flow cytometry. F. Graph of GFP^+^ *in vivo* MigR1 versus *Fli-1* pre-leukaemic thymocyte populations. Data are represented mean ± SEM; n = 4.G. Expression of *Fli-1* mRNA in thymocyte subsets (mean ± SEM). Sorted DN1, DN2, DN3, DN4, ISP CD8, DP, CD4 SP and CD8 SP thymocytes were subjected to QRT-PCR for *Fli-1*. DN data: n = 3;. DP and SP data: n = 2.

As *Fli-1* is expressed throughout T cell development it was hypothesized that retroviral *Fli-1* overexpression could potentially perturb T cell differentiation. In order to test this, we transduced foetal liver (FL) with control MigR1-GFP retrovirus or *Fli-1*-GFP retrovirus and reconstituted foetal thymic lobes. Fourteen days later, thymocytes were analysed by flow cytometry using antibodies to CD4 and CD8. It can be clearly seen that the *Fli-1* foetal thymic organ cultures (FTOC) demonstrated a significant inhibition of the DN to DP transition as evidenced by an increased percentage of DN and a decreased percentage of DP cells (Figure 1A–B). There was also a significant reduction in the percentage of the CD4^+^ SP population in *Fli-1* FTOCs. Finally, an expansion of the percentage of immature (TCRβ^−/lo^) CD8^+^ SP (ISP) cells was found in *Fli-1* FTOCs.

As *Fli-1* expression resulted in a retardation of the DN to DP transition we hypothesized that it may alter normal DN1–DN4 differentiation. Therefore, we cultured FL transduced with control MigR1 or *Fli-1* on OP9-DL1 cells for 8 days. Indeed, *Fli-1* significantly retarded development at the DN3 stage (Figure 1C–D). Additionally, *Fli-1* also significantly enhanced the differentiation of DN1 cells (Figure 1C–D).

*In vivo* validation of the FTOC results was performed by analysing lethally irradiated mice transplanted with FL or bone marrow (BM) cells transduced with control MigR1 or *Fli-1* retrovirus. After 10–12 weeks, thymocytes from MigR1 and *Fli-1* transplanted mice were assessed for CD4 and CD8 expression by flow cytometry. There was no difference in total (GFP^+^ and GFP^−^) thymocyte numbers between the control MigR1 and *Fli-1* overexpressing thymi (Figure S1). However, it was evident that *Fli-1* mice had a decreased percentage of CD4^+^ SP and a significantly increased percentage of CD8^+^ SP (Figure 1E–F). These results are consistent with the previous *Fli-1* FTOC data and demonstrate an acute effect of *Fli-1* overexpression on T cell development, which ultimately leads to an expansion of the percentage of CD8^+^ T cells and a reduction in the percentage of CD4^+^ T cells.

This led us to analyse *Fli-1* expression by QRT-PCR in normal DN1–4, ISP CD8, DP, CD4 SP and CD8 SP thymocytes (Figure 1G). The highest expression of *Fli-1* in immature thymocytes was in DN1 and DN2 whilst DN3 and DN4 had the lowest expression. As thymocytes matured, the levels of *Fli-1* increased such that DP, CD4 SP and CD8 SP had the highest levels of *Fli-1*. As expected, *Fli-1* overexpression seemed to mainly affect those T cell subpopulations that normally have low levels of *Fli-1*.

Given this data and that *Fli-1* activation had previously been shown to induce erythroleukaemia in BALB/c mice, it was hypothesized that *Fli-1* may induce T cell oncogenesis in C57BL/6 mice [1].

**Figure 2:**
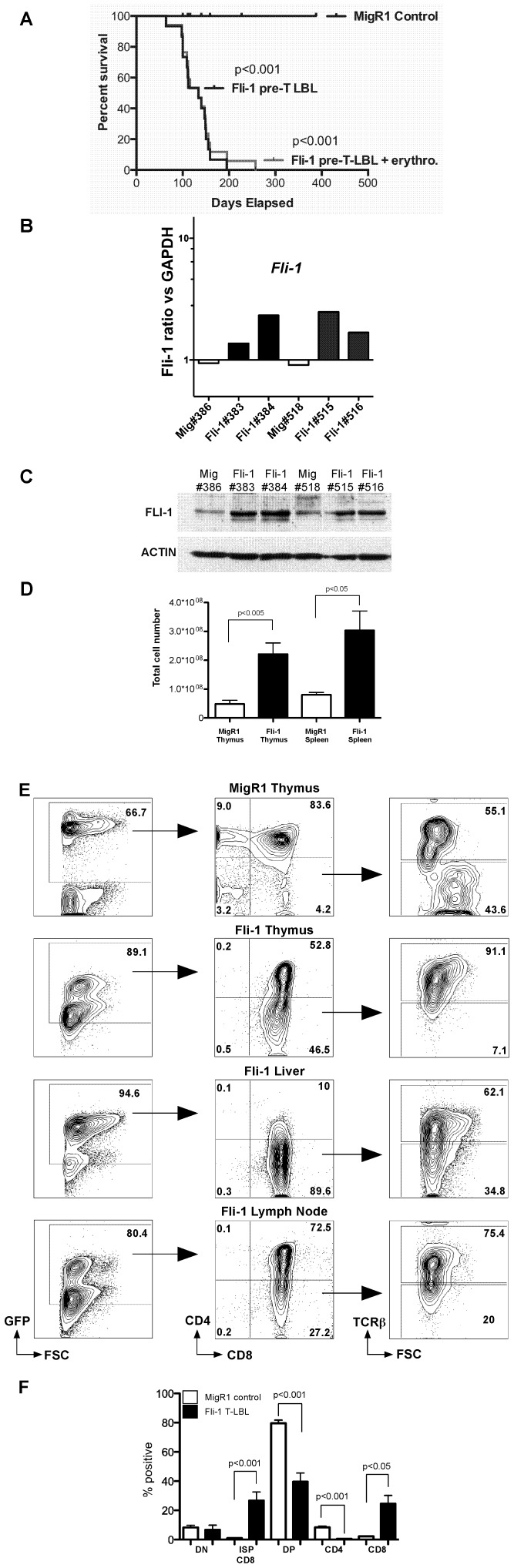
*Fli-1* overexpression induces pre-T LBL *in vivo*. BM or FL cells were transduced with MigR1 or *Fli-1* and used to transplant lethally irradiated mice. A. Kaplan-Meier survival curve of MigR1 (n = 8) versus *Fli-1* transplanted mice (n = 15). Two additional *Fli-1* mice developed an erythroleukaemia and were included in an additional Kaplan-Meier curve (Fli-1 pre-T LBL+eryth;gray curve n = 17). B. Q-PCR showing *Fli-1* mRNA levels of MigR1 control (Mig) or *Fli-1* thymocytes (*Fli-1*) C. Western Blot for FLI-1 protein of MigR1 control (Mig) or *Fli-1* thymocytes (*Fli-1*). ACTIN = loading control. D. Total cell numbers (GFP^+^ and GFP^−^) of thymus and spleen from lethally irradiated mice reconstituted with MigR1 and *Fli-1* expressing hematopoietic progenitors (mean ± SEM). E. MigR1 thymus and *Fli-1* thymus, liver and lymph node cells analysed for CD4, CD8 and TCRβ expression by flow cytometry. F. Graph of GFP^+^ DN, immature (TCRβ^lo^) single positive (ISP) CD8, DP, CD4 and mature TCRβ^hi^ CD8 SP thymocyte populations in MigR1 and *Fli-1* leukaemic mice. Data represented are mean ± SEM of 6 MigR1 mice and 13 *Fli-1* mice from 3 independent transplants.

As *Fli-1* overexpression severely perturbed T cell development after 10–12 weeks *in vivo* we monitored *Fli-1*-transplanted mice for leukaemia or lymphoma induction over an extended period. As hypothesized, *Fli-1* reconstituted mice presented with enlarged thymus, spleen and lymph nodes with a median onset of 111 days (Figure 2A-Kaplan-Meier Curve). Control MigR1 mice did not develop disease over the study period. However, two additional mice developed an erythroleukaemia (Figure 2A-grey curve). To confirm *Fli-1* overexpression we performed quantitative real-time PCR (Q-PCR) for mRNA expression on all mice (only six shown) and Western blot analysis for FLI-1 protein expression on two control MigR1 thymi and four *Fli-1* thymi that developed disease. This clearly demonstrated increased *Fi1* mRNA and FLI-1 protein expression in *Fli-1* thymi compared to the control MigR1 (Figure 2B–C).

Total cell numbers (GFP^+^ and GFP^−^) in *Fli-1* thymus and spleen were significantly higher than those of the MigR1 controls (Figure 2D). The *Fli-1* leukaemia phenotype was very similar to the preleukaemic phenotype with reduced CD4+, CD4^+^8^+^ and expanded CD8^+^ cells evident (Figure 2E). The CD4, CD8 and TCRβ phenotype of cells in the *Fli-1* thymus, liver and lymph node suggested that the disease was a T cell lymphoblastic leukaemia/lymphoma (pre-T LBL) [25]. However, there were two *Fli-1*-transplanted mice that had no surface TCRβ expression (Figure S2). *Fli-1* spleen and bone marrow were comparably abnormal (Figure S3).

*Fli-1* transplanted mice had significant percentage increases in both TCRβ^−/lo^ ISP CD8 and mature TCRβ^hi^ CD8 SP cells (Figure 2E). Additionally, the size of the *Fli-1* ISP CD8 cells in thymus, liver and lymph nodes was much smaller than control MigR1 ISP CD8s which are blasts; suggesting that *Fli-1* ISP CD8 cells were not leukaemic blasts (Figure 2E). *Fli-1* mice also had significant decreases in DP and CD4 SP cells, consistent with the FTOC data (Figs. 1A–B). Taken together, these results implied that *Fli-1* overexpression could induce a T cell malignancy resembling pre-T LBL and strongly suggest that *Fli-1* can act as a T cell oncogene.

### *Fli-1*-induced Pre-T LBL Perturbs Thymic, Splenic and Bone Marrow Architecture

**Figure 3:**
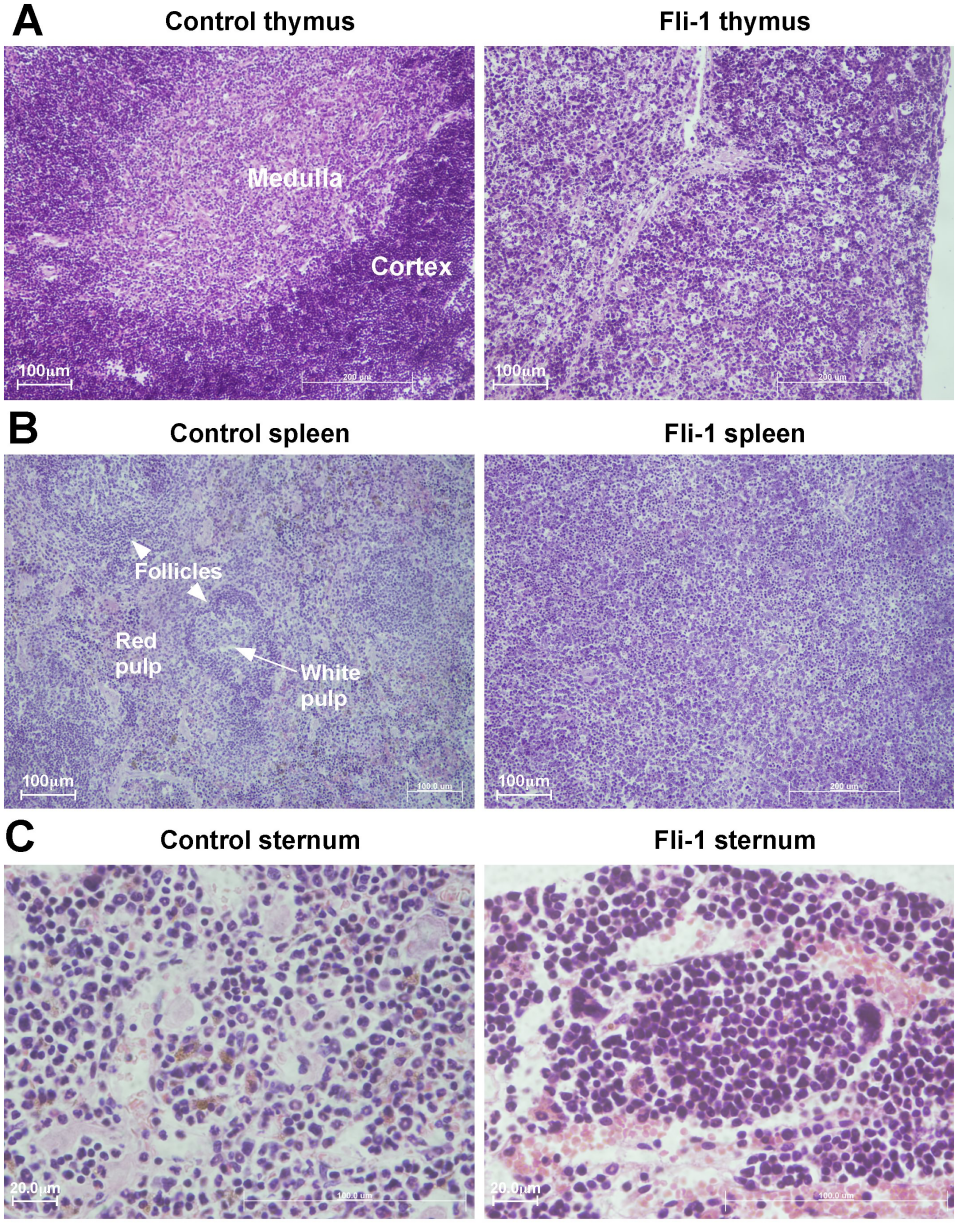
*Fli-1*-induced pre-T LBL perturbs thymic, splenic and bone marrow architecture. Representative tissue sections (Haematoxylin & Eosin stain) of MigR1 and *Fli-1* mice. MigR1 sections show normal organ architecture, whereas *Fli-1* sections show thymus and spleen architecture effaced by small infiltrating malignant lymphocytes also present in the *Fli-1* sternum. A. Thymus (x10 magnification). B. Spleen (x10 magnification). C. Sternum (x40 magnification).

Histology sections of MigR1 and *Fli-1* thymus, spleen, liver and sternum (BM) revealed a significant infiltration of small cells in all tissues of *Fli-1* transplanted mice (Figure 3). Specifically, the *Fli-1* thymus had lost its normal cortical-medullary demarcation and was completely permeated with malignant T cells (Figure 3A). The *Fli-1* spleen had no B cell follicles whilst the MigR1 control spleen did (Figure 3B. arrowheads). Finally, the bone marrow of the *Fli-1* sternum was filled with uniform-sized cells in comparison to the more sparse heterogeneity of the bone marrow cells from the MigR1 control (Figure 3C). These data are consistent with the flow cytometry results demonstrating that *Fli-1* transplanted mice had leukaemic cells throughout the thymus, spleen, lymph node, liver and bone marrow.

### *Fli-1* Pre-T LBL can be Transplanted to Secondary Recipients, is Integration Site Independent and Oligoclonal

**Figure 4:**
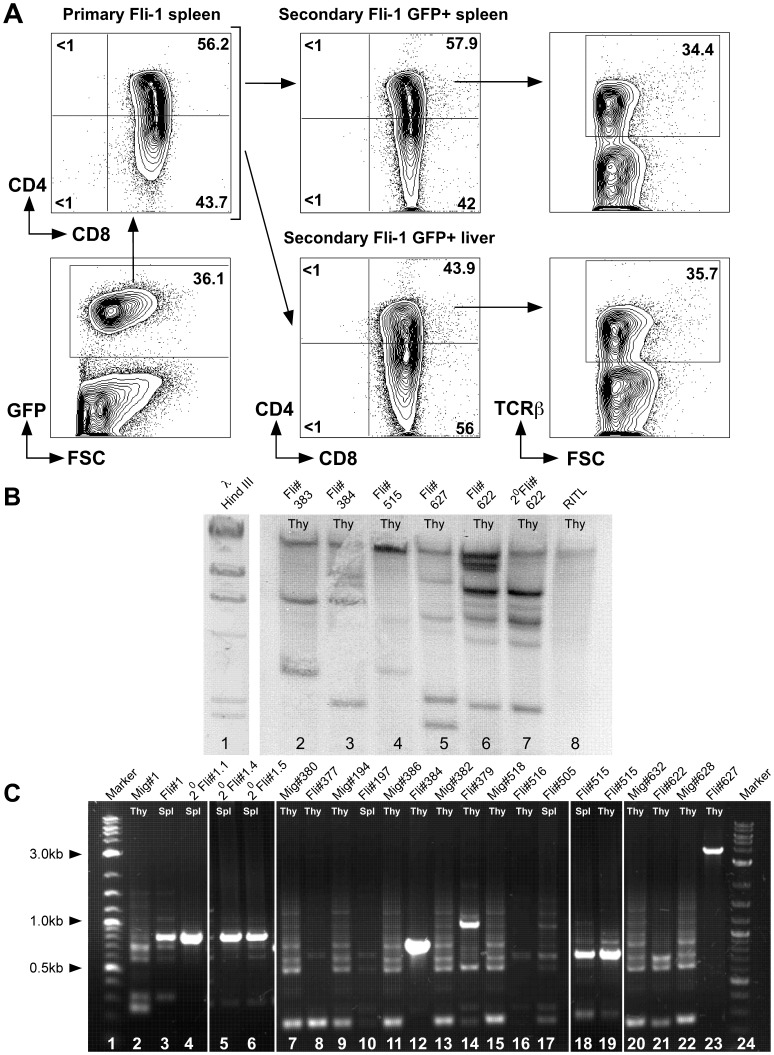
*Fli-1* pre-T LBL can be transplanted to secondary recipients, is not due to retroviral insertion site and is oligoclonal. A. Spleen cells from a mouse with *Fli-1* leukaemia were injected into 4 sublethally irradiated recipients, which subsequently developed disease around 3 weeks post-transplant. Three additional transplants into 11 unirradiated mice gave similar results. Single cell suspensions from spleen and liver were analysed for CD4, CD8 and TCRβ expression by flow cytometry. Data representative of 4 independent transplants. B. Southern blot of total thymocyte (thy) genomic DNA from mice transplanted with *Fli-1* transduced bone marrow showing multiple different retroviral integration sites in *Fli-1* leukaemia. *Eco*RI digested DNA was probed with a DIG-labelled IRES-GFP fragment. Lane 1: λ Hind III marker, 2–6: primary *Fli-1* thymi as indicated, 7: secondary from *Fli-1* #622, 8: mouse #625 Radiation-Induced Thymic Lymphoma (RITL). Data representative of three independent experiments. C. TCRβ VDJ rearrangement as assessed by PCR showing normal VDJ rearrangement in MigR1 control mice (7 bands: Vβ-Jβ2.1–2.8) and oligoclonal rearrangement in *Fli-1* mice (1–4 bands). Lanes 1 & 24: Marker, 2–23: MigR1 control (Mig) or primary and secondary (2°) *Fli-1* thymocytes (Thy) or spleen cells (Spl) as indicated. 23: *Fli-1* #627 is TCRβ^−^.

To ascertain if *Fli-1* pre-T LBL could be transferred to secondary recipients, 3×10^6^ spleen cells from a *Fli-1* mouse were injected intravenously into sublethally irradiated recipients. All mice became hunched, moribund and had ruffled fur after 20–21 days. Therefore, spleens and livers from these mice were analysed for expression of CD4, CD8 and TCRβ by flow cytometry. All 4 mice demonstrated a similar CD4^+^8^+^ population and CD8^+^ SP population as the primary mouse (Figure 4A). These data reveal that the *Fli-1*-induced pre-T LBL is transplantable.

To preclude that site specific integration effects could be responsible for the observed pre-T LBL development, a genomic Southern blot of both primary and secondary pre-T LBLs was undertaken with a GFP specific probe. The results unequivocally demonstrate that each individual pre-T LBL had different multiple integration sites and as such the observed pre-T LBL is due to aberrant FLI-1 expression (Figure 4B). Additionally, we sequenced integration sites in 5 tumours and could find no association with known oncogenes (Table S1).

Interestingly, analysis of TCRβ rearrangements revealed that most *Fli-1* pre-T LBL possessed one dominant clone but were oligoclonal (Figure 4C). The exceptions to this were those that did not express TCRβ (Figure 4C, lanes 16 & 23). As expected, identical TCRβ rearrangements were found in the thymus and spleen of the same *Fli-1* transplanted mice (Figure 4C, lanes 18–19) and in primary and secondary *Fli-1* transplanted mice (Figure 4C, lanes 3–6).

### No Increase in Pro-survival *Bcl-2* Family mRNAs in *Fli-1* Pre-T LBL

**Figure 5:**
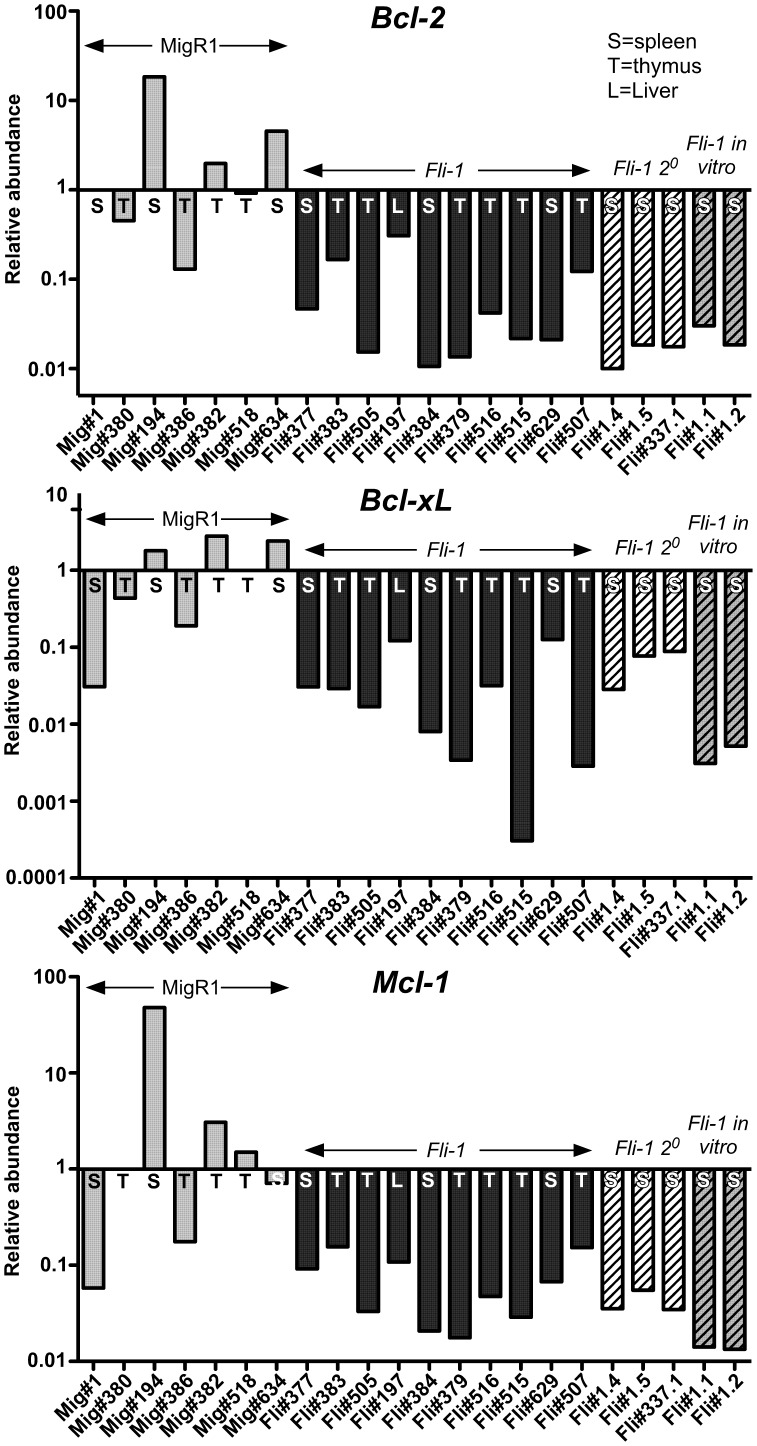
No upregulation of pro-survival *Bcl-2* family mRNA in *Fli-1* pre-T LBL. RNA was isolated from MigR1 control (Mig), *Fli-1* primary, secondary (2°) and *in vitro Fli-1* pre-T LBL cells from the thymus, spleen or liver and cDNA was subjected to Q-PCR for the expression of *Bcl-2*, *Bcl-xL* and *Mcl-1* mRNA. Samples were normalized to GAPDH levels and are shown on a log scale relative to the median MigR1 value, which was set to 1. T = thymus, S = spleen, L = liver.

*Fli-1* has been demonstrated to upregulate *Bcl-2* in erythroleukaemia [26]. Therefore, we analysed primary and secondary *Fli-1* leukaemias and MigR1 controls for *Bcl-2*, *Bcl-xL* and *Mcl-1* mRNA expression by Q-PCR (Figure 5). Surprisingly, all *Fli-1* pre-T LBLs, whether primary, secondary or grown *in vitro* had lower levels of *Bcl-2* family members, *Bcl-2*, *Bcl-xL* and *Mcl-1* (Figure 5). These data show that overexpression of the pro-survival *Bcl-2* family members are unlikely to be involved in the induction of *Fli-1* pre-T LBL.

### Intracellular NOTCH1 is Upregulated in *Fli-1* Pre-T LBL

**Figure 6:**
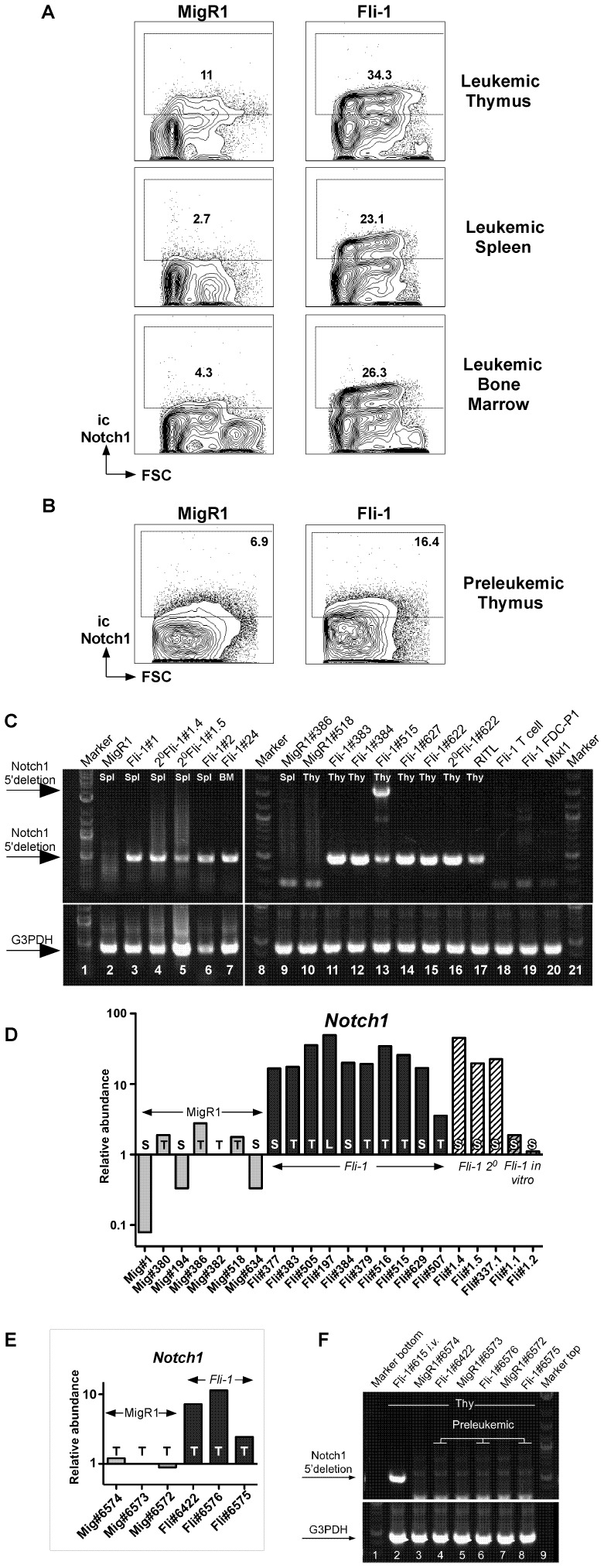
*Fli-1* pre-T LBL cells demonstrate elevated levels of intracellular NOTCH1 protein and carry *Notch1* mutations that result in ligand-independent transcriptional activation. A. *Fli-1* thymus spleen and bone marrow examined by flow cytometry for intracellular NOTCH1. Data is representative of three independent experiments. B. Intracellular NOTCH1 expression was analysed by flow cytometry in *Fli-1* preleukaemic thymi in comparison to the MigR1 control 6 weeks post transplant. Data is representative of three independent experiments. C. *Fli-1* pre-T LBL possess 5′ *Notch1* deletions detectable by PCR. Primers flanking the 5′ *Notch1* deletion site generate a small 485 bp PCR fragment of genomic DNA in *Fli-1* tumours, which is absent in control cells where the same primers span a 12,251 bp region of undeleted WT DNA. Lanes 1, 8 & 21∶2-Log DNA ladder, 2–7 and 9–17: MigR1 control or primary and secondary (2°) *Fli-1* spleen cells (Spl), bone marrow (BM) or thymocytes (Thy) as indicated. 17; radiation-induced thymic lymphoma (RITL), 18: *Fli-1* transduced T cell precursors grown on OP9-DL1 cells, 19: *Fli-1*-transduced FDC-P1 cells, 20: *Mixl1*-transduced leukaemic BM cells. G3PDH: positive gDNA PCR control. D. *Notch1* mRNA upregulation in leukaemic *Fli-1* mice. Q-PCR of MigR1 control (Mig), *Fli-1* primary, secondary (2°) and *in vitro Fli-1* pre-T LBL cells from the thymus, spleen or liver. Samples and analysis as in Fig. 5. E. *Notch1* mRNA upregulation in preleukaemic *Fli-1* mice (see Fig.1 F and 6B). F. Absence of 5′ *Notch1* deletions in preleukaemic *Fli-1* mice. Lane 1 & 9∶2-Log ladder, lane 2: positive 5′ *Notch1* deletion control, 3–8: MigR1 control and preleukaemic *Fli-1* thymocytes as indicated.

*NOTCH1* plays a central role in T cell development and NOTCH1 mutations have now been detected not only in around 55% of all human T-ALL but also in a high fraction of murine pre-T LBL [22], [23], [24], [27], [28]. Therefore, we chose to analyse tissues from both MigR1 control and *Fli-1* tumour cells for the presence of intracellular NOTCH1 protein by flow cytometry. Firstly, the amount of intracellular NOTCH1 staining seen in the *Fli-1* thymus was significantly greater than the MigR1 control (Figure 6A) (p<0.05). All *Fli-1* tissues examined expressed more intracellular NOTCH1 than the corresponding MigR1 control (Figure 6A). Additionally, there was at least a doubling of intracellular NOTCH1 in all preleukaemic *Fli-1* thymi tested at 6 weeks post-transplant compared to MigR1 control thymi (Figure 6B).

Until recently mutations in the PEST domain were thought to be the most common *Notch1* mutation in murine pre-T LBL [27]. Since then it has been discovered that 5′ *Notch1* deletions, resulting in ligand-independent activation, are far more frequent [23], [24]. When we sequenced the PEST domain of *Fli-1* tumours, only four out of eight primary samples had mutations (Table S2). We therefore analysed MigR1 control and *Fli-1* tumour cells for the presence of 5′ *Notch1* deletions. It was found that all primary and secondary *Fli-1* pre-T LBL as well as a radiation-induced thymic lymphoma contained type 1 5′ *Notch1* deletions [23] (Figure 6C). *Fli-1* transduced T cell precursors and FDC-P1 cells and *Mixl* transduced myeloid cells were all negative (Figure 6C). Additionally, sequencing of the flanking regions of the 5′ *Notch1* deletions showed evidence of different RAG dependent type 1 rearrangements in each pre-T LBL including one rearrangement at a novel RSS site (Table S3) [23].

The presence of these 5′ *Notch1* deletions also explains the observed upregulation of *Notch1* on the mRNA level (Figure 6D), since they confer ligand independent transcriptional activation from internal sites in or close to exon 25. However, preleukaemic *Fli-1* thymi also showed *Notch1* mRNA upregulation (Figure 6E) in the absence of 5′ *Notch1* deletions (Figure 6F), suggesting other mechanisms of *Notch1* activation might play a role in the early stages of *Fli-1* induced pre-T LBL.

Taken together, these results demonstrate that *Fli-1* overexpression induced a pre-T LBL in mice. This malignancy was associated with upregulation of *Notch1* mRNA and protein before frank leukaemia was observed. 5′ *Notch1* deletions were found in all *Fli-1* leukaemic cells.

## Discussion

*Fli-1* was initially identified as an insertion site in erythroleukaemia in BALB/c mice and subsequently shown to be required for the development of megakaryocytes [1], [3]. *Fli-1* has also been found to control myeloid and B cell development [4], [29]. However, the data presented here for the first time, demonstrate that *Fli-1* also plays a role in T cell development. *Fli-1* overexpression leads to a block in the DN3 to DN4 transition with a subsequent inhibition of the DN to DP transition. The *Fli-1*-induced DN3 arrest is consistent with endogenous *Fli-1* downregulation at the DN2 stage as enforced *Fli-1* expression in DN3 would increase FLI-1 levels and inhibit progression to DN4.

*Fli-1* overexpression *in vivo* eventually causes a pre-T LBL in C57BL/6 mice, outcompeting all other lineages. Usually, it is thought an acceleration of the DN to DP transition is leukaemogenic as in the case of activated *Ras* or *Ikaros* deficient cells [30], [31], [32]. However, a delay of the DN to DP transition as seen in *Lck* and *Scl/Lmo* transgenic and E2A deficient mice can also give rise to T cell lymphoma [33], [34], [35]. Clearly, any deregulation of this expansionary phase of T cell development driven by the pre-TCR can have dire consequences.

The detection of atypical small TCRβ^lo^ ISP CD8 in *Fli-1* thymus, spleen, liver, lymph node and bone marrow is similar to a conditional *Scl* transgenic that developed T cell malignancy [36]. In that report, ISP CD8 cells were derived from DP rather than DN cells. Intriguingly, both *Fli-1* and *Scl* are basic helix-loop-helix transcription factors that are involved in early hematopoietic specification from the hemangioblast [37].

One of the main mechanisms proposed for *Fli-1* erythroblast survival in erythroleukaemia and increased B cell survival in *H2-K^K^-Fli-1* mice is upregulation of *Bcl-2* [7]. Increased *Bcl-2* has also been shown to play a major role in a zebrafish model of pre-T LBL [38]. However, we found no evidence of *Bcl-2*, *Bcl-xL* or *Mcl-1* mRNA upregulation *in Fli-*1 pre-T LBL. On the contrary, expression of all pro-survival *Bcl-2* family members appeared downregulated; a phenotype more reminiscent of E2A deficient lymphomas [39].

This suggested the involvement of another, possibly more T cell specific signalling pathway. We decided to focus on *Notch1* since activating mutations of *Notch1* are common to both human T-ALL and mouse pre-T LBL [40]. Intracellular NOTCH1 protein was indeed increased in all *Fli-1* pre-T LBL cells and was associated with 5′ *Notch1* deletions and PEST mutations. As expected, all *Fli-1* pre-T LBL also expressed high levels of *Notch1* mRNA, but the elevated *Notch1* mRNA level in preleukaemic *Fli-1* mice was unforeseen. As *Fli-1* is expressed dynamically throughout the DN to DP transition [6], ectopic *Fli-1* expression may alter the balance of a number of pre-TCR transcription factors leading to ectopic signalling of a number of genes including *Notch1*. SCL, for example, heterodimerises with E2A and HEB and downregulates their expression [41], [42]. E2A and NOTCH1 directly regulate *Notch1* transcription in pre-β-selected thymocytes and *Notch1* activation is one of the early events in SCL-induced leukaemogenesis [36], [43]. NOTCH1 also feeds back into this pathway by inducing SCL degradation [44]. A similar role has been proposed for ETS1 and E2A in T cell development and FLI-1 and E2A in B cell development [45], [46]. Alternatively, although not mutually exclusive, *Fli-1* overexpression may create a preleukaemic environment where *Notch1* mutations accrue much more readily than normal [34]. We have shown that *Fli-1* cells accumulate at the DN3 stage and studies have shown that the *Notch1* and pre-TCR signalling pathways cooperate at this stage during T cell development and transformation [43], [47]. Leukaemic transformation might happen at a later stage as evidenced by the presence of 5′ *Notch1* deletions in pre-T LBL, but not in preleukaemic cells. These specific *Notch1* deletions are introduced by inappropriate *Rag2* dependent recombination (Table S3) and *Rag2* expression is highest in small resting DPs as seen in pre-T LBL [23], [48]. Indeed, Fli-1 binding sites have been found in the Notch1 promoter in haemopoietic cells [49](Table S2).

The role of NOTCH1 and *NOTCH1* gain-of-function mutations in human T-ALL (including activating deletions) as well as its use as a target in the treatment of T-ALL has been well documented [50], [51], [52]. There is also ample evidence for abnormal *FLI-1* expression in human haematological malignancies including T cell malignancies (Figure S4, [53], [54]). We have shown that *Fli-1* appears to collaborate with *Notch1* to induce pre-T LBL.

It therefore could be envisaged that the synergistic use of *Fli-*1 inhibitors and γ-secretase inhibitors would provide a potent therapeutic combination for human T-ALL. Given that FLI-1 inhibitors have already been identified using human Ewing’s-sarcoma and erythroleukaemic cell lines, the *Fli-1* mouse model developed here should be informative in elucidating pathways critical for inhibiting *Fli-1-*induced T cell proliferation [55], [56].

## Supporting Information

Figure S1 **Total thymocyte numbers (GFP^+^ and GFP^−^) for mice transplanted with either MigR1 control or** ***Fli-1*** **analysed at 10–12 weeks post-transplant (MigR1 GFP%: 84.2, 41,1 and 85;** ***Fli-1*** **GFP%: 83.4, 86.2 and 76.8).**
(TIF)

Figure S2 **Thymic phenotypes of** ***Fli-1*** **T-LBL.** Thymi from 9 *Fli-1* mice were analysed by flow cytometry for expression of CD4 and C8. Mouse numbers are indicated at the top of each plot. Mouse #516 is an example of a TCR- *Fli-1* tumour. The remainder of the mice have TCRβ on their surface.
(TIF)

Figure S3 ***Fli-1*** **spleen and bone marrow have the same phenotype as** ***Fli-1*** **thymus, liver and lymph node.** *Fli-1* spleen and bone marrow were analysed for CD4, CD8 and TCRβ expression by flow cytometry. Data representative of 6 MigR1 mice and 13 *Fli-1* mice from at least 3 independent transplants.
(TIF)

Figure S4 **Human** ***FLI-1*** **and** ***NOTCH1*** **mRNA expression levels across a large number of healthy (light grey) and pathological (dark grey) human tissues retrieved from the IST database system developed by MediSapiens Ltd.** The current version (4.3) contains 20,218 human tissue and cell line samples analysed by Affimetrix gene expression microarrays. (Kiplinen et al. Genome Biol. 2008;9 (9): R139. Autio et al. BMC Bioinformatics. 2009 Jan 30; 10 Suppl 1:S24).
(TIF)

Table S1 Insertion sites in *Fli-1* tumours. A. Number of insertion sites in *Fli-1* tumours identified by Southern Blot and LM-PCR and cloned for sequence analysis. B. Insertion site analysis of *Fli-1* integrations: position of *Fli-1* integration (<: insertion downstream or >: upstream of indicated position) and genes identified in or near the integration site.
(DOC)

Table S2 Notch1 PEST mutations. The PEST domain of murine *Notch1* was amplified from genomic DNA isolated from total thymus or spleen and gel purified and sequenced. ^1^ position in GenBank sequence no. AL73541.11. RITL: Radiation Induced Thymic Lymphoma.
(DOC)

Table S3 5′ *Notch1* deletion sequences. Rearrangements in *Notch1* deduced from sequencing of PCR products shown in figure 6C. RAG dependent recombination occurred between the −8191 RSS and the +3575 RSS described in reference 24 or a newly identified −7090 RSS and the +3575 RSS. The −7090 RSS was determined using the Recombination Signal Sequence Site http://www.itb.cnr.it/rss/. (RIC score of −45.2 compared to −41.2 for the −8191 RSS and −66.3 for the +3575 RSS). RSS: cryptic RAG signal sequences and their location relative to the position of the ATG start codon in exon 1 of *Notch1*. GL: sequence of the germ line DNA flanking the breakpoints. ^1 2^: Indicate two different clones within the same tumour.
(DOC)
