# Supplementary material for: Fli-1 Overexpression in Hematopoietic Progenitors Deregulates T Cell Development and Induces Pre-T Cell Lymphoblastic Leukaemia/Lymphoma
Source: PLoS One. 2013 May 7;8(5):e62346. doi: 10.1371/journal.pone.0062346 (PMC3646842; doi:10.1371/journal.pone.0062346)
Supplement: Table S1 — Insertion sites in Fli-1 tumours. A. Number of insertion sites in Fli-1 tumours identified by Southern Blot and LM-PCR and cloned for sequence analysis. B. Insertion site analysis of Fli-1 integrations: position of Fli-1 integration (<: insertion downstream or >: upstream of indicated position) and genes identified in or near the integration site. (DOC) [file pone.0062346.s005.doc]

**Table S1A**. Number of insertion sites identified in *Fli-1* tumours and cloned for analysis.

| **Tumour** | **Number of bands identified by Southern Blot** | **Number of bands identified by LM-PCR** | **Number of LM-PCR bands cloned and sequenced** |
| --- | --- | --- | --- |
| Fli-1 #383 | 2 | 2 | 2 |
| Fli-1 #384 | 2 | 1 | 1 |
| Fli-1 #379 | ND | 3 | 2 |
| Fli-1 #515 | 2 | 1 | 1 |
| Fli-1 #507 | ND | 2 | 2 |

ND: not determined.

**Table S1B**. Insertion site analysis of *Fli-1* integrations.

| **Tumour** | **Insertion site position Locus (Chromosome #)** | **Features in or flanking this part of the subject sequence** |
| --- | --- | --- |
| Fli-1 #383 ins. 1 | <8707002 [NT_039474.7](http://www.ncbi.nlm.nih.gov/mapview/maps.cgi?maps=blast_set&db=alt_contig+ref_contig+rna&na=1&gnl=ref|NT_039474.7|&gi=149260095&term=149260095%5Bgi%5D&taxid=10090&RID=ZGHDP3JF01S&QUERY_NUMBER=1&log$=nuclalign) (9) | [Coro2b](http://www.ncbi.nlm.nih.gov/nucleotide/82923584?report=gbwithparts&from=56103952&to=56211014&RID=ZGHDP3JF01S) |
| Fli-1 #383 ins. 2 | <54192019 [NT_078575.6](http://www.ncbi.nlm.nih.gov/mapview/maps.cgi?maps=blast_set&db=alt_contig+ref_contig+rna&na=1&gnl=ref|NT_078575.6|&gi=149259126&term=149259126%5Bgi%5D&taxid=10090&RID=ZGKZG3CD016&QUERY_NUMBER=1&log$=nuclalign) (8) | [240851 bp at 5' side: Irf2bp2](http://www.ncbi.nlm.nih.gov/nucleotide/149259126?report=gbwithparts&from=53948367&to=53950690&RID=ZGKZG3CD016)  [100251 bp at 3' side: Tomm20](http://www.ncbi.nlm.nih.gov/nucleotide/149259126?report=gbwithparts&from=54292269&to=54302978&RID=ZGKZG3CD016) |
| Fli-1 #384 ins. 1 | >19334398 [NT_166318.1](http://www.ncbi.nlm.nih.gov/mapview/maps.cgi?maps=blast_set&db=alt_contig+ref_contig+rna&na=1&gnl=ref|NT_166318.1|&gi=149263585&term=149263585%5Bgi%5D&taxid=10090&RID=ZGMYNSWY014&QUERY_NUMBER=1&log$=nuclalign)(12) | [478495 bp at 5' side: hyp. prot. LOC100503537](http://www.ncbi.nlm.nih.gov/nucleotide/149263585?report=gbwithparts&from=18844748&to=18855903&RID=ZGMYNSWY014)  [345331 bp at 3' side: hyp. prot. LOC73160](http://www.ncbi.nlm.nih.gov/nucleotide/149263585?report=gbwithparts&from=19679974&to=19680336&RID=ZGMYNSWY014) |
| Fli-1 #379 ins. 1 | >6124602 [NT_039580.7](http://www.ncbi.nlm.nih.gov/mapview/maps.cgi?maps=blast_set&db=alt_contig+ref_contig+rna&na=1&gnl=ref|NT_039580.7|&gi=149264241&term=149264241%5Bgi%5D&taxid=10090&RID=ZGNERBY8016&QUERY_NUMBER=1&log$=nuclalign) (13) | [303752 bp at 5' side: Atxn1](http://www.ncbi.nlm.nih.gov/nucleotide/149264241?report=gbwithparts&from=5809488&to=5820850&RID=ZGNERBY8016)  [92477 bp at 3' side: hyp. prot. LOC76000](http://www.ncbi.nlm.nih.gov/nucleotide/149264241?report=gbwithparts&from=6217711&to=6218654&RID=ZGNERBY8016) |
| Fli-1 #379 ins. 2 | <41867207 [NT_096135.5](http://www.ncbi.nlm.nih.gov/mapview/maps.cgi?maps=blast_set&db=alt_contig+ref_contig+rna&na=1&gnl=ref|NT_096135.5|&gi=149262021&term=149262021%5Bgi%5D&taxid=10090&RID=ZGR1K94B01S&QUERY_NUMBER=1&log$=nuclalign) (11) | Abr |
| Fli-1 #515 ins. 1 | <53071690 [NT_096135.5](http://www.ncbi.nlm.nih.gov/mapview/maps.cgi?maps=blast_set&db=alt_contig+ref_contig+rna&na=1&gnl=ref|NT_096135.5|&gi=149262021&term=149262021%5Bgi%5D&taxid=10090&RID=ZGT5BJ5G014&QUERY_NUMBER=1&log$=nuclalign) (11) | [9758 bp at 5' side: Supt4h1](http://www.ncbi.nlm.nih.gov/nucleotide/149262021?report=gbwithparts&from=53055980&to=53061693&RID=ZGT5BJ5G014)  [8092 bp at 3' side: Bzrap1](http://www.ncbi.nlm.nih.gov/nucleotide/149262021?report=gbwithparts&from=53079782&to=53101069&RID=ZGT5BJ5G014) |
| Fli-1 #507 ins. 1 | <14494365 [NT_165773.2](http://www.ncbi.nlm.nih.gov/mapview/maps.cgi?maps=blast_set&db=alt_contig+ref_contig+rna&na=1&gnl=ref|NT_165773.2|&gi=149262584&term=149262584%5Bgi%5D&taxid=10090&RID=ZK5AB1XC01S&QUERY_NUMBER=1&log$=nuclalign) (11) | [Plcd3](http://www.ncbi.nlm.nih.gov/nucleotide/149262584?report=gbwithparts&from=14473230&to=14503919&RID=ZK5AB1XC01S) |
| Fli-1 #507 ins. 2 | <797950 [NT_166302.1](http://www.ncbi.nlm.nih.gov/mapview/maps.cgi?maps=blast_set&db=alt_contig+ref_contig+rna&na=1&gnl=ref|NT_166302.1|&gi=149254745&term=149254745%5Bgi%5D&taxid=10090&RID=ZGTPSV7W01N&QUERY_NUMBER=1&log$=nuclalign) (5) | [113127 bp at 5' side: Nptx2](http://www.ncbi.nlm.nih.gov/nucleotide/149254745?report=gbwithparts&from=674493&to=684823&RID=ZGTPSV7W01N)  [53775 bp at 3' side: Rps29](http://www.ncbi.nlm.nih.gov/nucleotide/149254745?report=gbwithparts&from=852056&to=852226&RID=ZGTPSV7W01N) |

Table S1. Insertion sites in *Fli-1* tumours. A. Number of insertion sites in *Fli-1* tumours identified by Southern Blot and LM-PCR and cloned for sequence analysis. B. Site analysis of *Fli-1* integrations and the genes identified. <: insertion downstream or >: upstream of indicated position.
