# Supplementary material for: Fli-1 Overexpression in Hematopoietic Progenitors Deregulates T Cell Development and Induces Pre-T Cell Lymphoblastic Leukaemia/Lymphoma
Source: PLoS One. 2013 May 7;8(5):e62346. doi: 10.1371/journal.pone.0062346 (PMC3646842; doi:10.1371/journal.pone.0062346)
Supplement: Table S2 — Notch1 PEST mutations. The PEST domain of murine Notch1 was amplified from genomic DNA isolated from total thymus or spleen and gel purified and sequenced. 1 position in GenBank sequence no. AL73541.11. RITL: Radiation Induced Thymic Lymphoma. (DOC) [file pone.0062346.s006.doc]

**Table S2**

Notch1 PEST mutations.

| ***Fli-1* tumour** | **Mutation** | **Position^1^** |
| --- | --- | --- |
| Fli-1 #1 | None |  |
| 2º Fli-1 #1.1 | None |  |
| 2º Fli-1 #1.2 | None |  |
| Fli-1 #2 | None |  |
| Fli-1 #24 | None |  |
| Fli-1 #383 | tgcacaccattct>CT | 68481 |
| Fli-1 #384 | atgtacaaccgctgggccccagcagtc>TC | 668448 |
| Fli-1 #515 | G>GG | 66305 |
| Fli-1 #627 | None |  |
| Fli-1 #622 | CG>CCC | 66391 |
| 2º Fli-1 #622 | CG>CCC | 66391 |
| RITL | G>GAAACTAGG | 66393 |

^1^ position in GenBank sequence no. AL73541.11

RITL Radiation Induced Thymic Lymphoma
