# Supplementary material for: Fli-1 Overexpression in Hematopoietic Progenitors Deregulates T Cell Development and Induces Pre-T Cell Lymphoblastic Leukaemia/Lymphoma
Source: PLoS One. 2013 May 7;8(5):e62346. doi: 10.1371/journal.pone.0062346 (PMC3646842; doi:10.1371/journal.pone.0062346)
Supplement: Table S3 — 5′ Notch1 deletion sequences. Rearrangements in Notch1 deduced from sequencing of PCR products shown in figure 6C. RAG dependent recombination occurred between the −8191 RSS and the +3575 RSS described in reference 24 or a newly identified −7090 RSS and the +3575 RSS. The −7090 RSS was determined using the Recombination Signal Sequence Site http://www.itb.cnr.it/rss/. (RIC score of −45.2 compared to −41.2 for the −8191 RSS and −66.3 for the +3575 RSS). RSS: cryptic RAG signal sequences and their location relative to the position of the ATG start codon in exon 1 of Notch1. GL: sequence of the germ line DNA flanking the breakpoints. 1 2: Indicate two different clones within the same tumour. (DOC) [file pone.0062346.s007.doc]

**Table S3**

5’ *Notch1* deletion sequences.

**-8191 RSS** **+3575 RSS**

**GL** GATGCCC**CACCTCA(12bp)ACATGAAGG CTTTATAGC(23bp)CACTGTGTAGGCT**

Fli-1 #383 GATGCCC**......................GTA......................TAGGCT**

Fli-1 #384 GAT**..........................GAA......................TAGGCT**

Fli-1 #515^1^ GATGC**.....................ATGGGGGAG....................AGGCT**

Fli-1 #627^1^ GATGCCC.**.....................GAA......................TAGGCT**

Fli-1 #627^2^ GATGCCC.**....................GGGGAG.....................AGGCT**

Fli-1 #622 GA...**.......................GCCCC........................GCT**

2ºFli-1 #622 GA...**.......................GCCCC........................GCT**

**-7090 RSS** **+3575 RSS**

**GL** CCTTCCA**CACAGTG(12bp)TCAACCTTC CTTTATAGC(23bp)CACTGTGTAGGCT**

Fli-1 #515^2^ CC**...........................CGTA.....................TAGGCT**

RSS: cryptic RAG signal sequences and their location relative to the position of the ATG start codon in exon 1 of *Notch1*. GL: sequence of the germ line DNA flanking the breakpoints. ^1 2^: Indicate two different clones within the same tumour.

Rearrangements in *Notch1* deduced from sequencing of PCR products shown in figure 6C. RAG dependent recombination occurred between the -8191 RSS and the +3575 RSS described in reference 24 or a newly identified -7090 RSS and the +3575 RSS. The -7090 RSS was determined using the Recombination Signal Sequence Site <http://www.itb.cnr.it/rss/>. (RIC score of -45.2 compared to -41.2 for the -8191 RSS and -66.3 for the +3575 RSS).
